# Supplementary material for: Causal Relationship between Aging and Anorexia Nervosa: A White-Matter-Microstructure-Mediated Mendelian Randomization Analysis
Source: Biomedicines. 2024 Aug 16;12(8):1874. doi: 10.3390/biomedicines12081874 (PMC11351342; doi:10.3390/biomedicines12081874)
Supplement: Supplementary file 1 [file biomedicines-12-01874-s001.zip › Supplementary Figure/Figure S8/Leaveoneout plot of MR analysis for TL to ubm-b-1653.pdf]

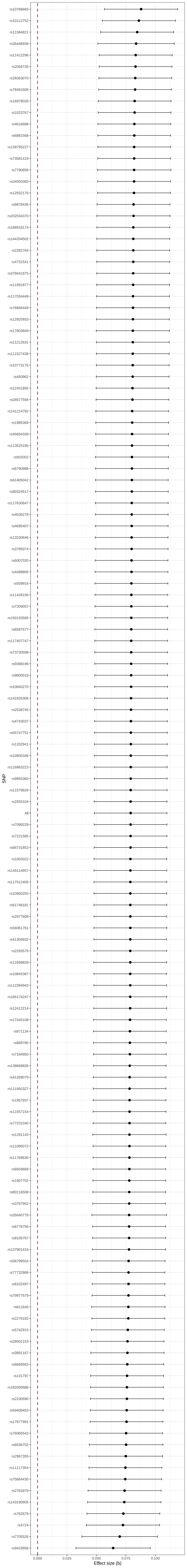

rs10768683
rs10112752
rs11584821
rs35446936
rs11412296
rs2056726
rs28363070
rs78491606
rs16978028
rs1023767
rs4616688
rs6881568
rs139795227
rs73581419
rs7790856
rs34550383
rs12932179
rs9878436
rs202034370
rs188918174
rs144204502
rs2282764
rs4731541
rs376641875
rs11991877
rs117034449
rs76666449
rs12925933
rs17803849
rs11212631
rs111527438
rs10773176
rs450962
rs12451892
rs28577594
rs141214782
rs1985369
rs45604339
rs113525195
rs932002
rs6790988
rs61405042
rs80324517
rs117630647
rs4530278
rs4695407
rs13230646
rs3785074
rs6007020
rs4498805
rs939916
rs11426156
rs7209057
rs150150565
rs6587577
rs117407747
rs73730598
rs9398196
rs9600019
rs10640270
rs142426306
rs2538745
rs4743037
rs55747751
rs1332941
rs10605346
rs116863223
rs9955360
rs11579626
rs2555104
All
rs7099229
rs7221585
rs66731853
rs1003322
rs145114957
rs117512405
rs10905255
rs61748181
rs2977608
rs56061761
rs41304832
rs2293579
rs11699829
rs10845387
rs112394943
rs185174247
rs12412214
rs17445108
rs871134
rs869785
rs7164950
rs139669835
rs41269078
rs111950327
rs1957937
rs11557154
rs77231040
rs1291143
rs11085072
rs11769630
rs6659669
rs1907702
rs80116508
rs3767952
rs35640778
rs6776756
rs8105767
rs137901416
rs56799554
rs77732866
rs8102497
rs79977579
rs611646
rs2276182
rs5742915
rs28502153
rs3891167
rs6669563
rs131797
rs182059586
rs2230590
rs59409453
rs17677991
rs76065543
rs6536702
rs2967355
rs11117354
rs75664430
rs2763979
rs143190905
rs762679
rs4724
rs7705526
rs9419958
SNP
0.000
0.025
0.050
0.075
0.100
Effect size (b)
